# Editorial: Public Health Data Challenges of the COVID-19 pandemic: A Sisyphean task!

**DOI:** 10.3389/fpubh.2022.1010055

**Published:** 2022-10-13

**Authors:** Carla Sofia Farinha, Paulo Jorge Nogueira, Rodrigo Feteira-Santos, Andreia Silva Costa

**Affiliations:** ^1^Statistics Portugal, Presidência dop Conselho de Ministros, Lisboa, Portugal; ^2^Center for Environmental and Sustainability Research (CENSE), NOVA School of Science and Technology (FCT), NOVA University of Lisbon, Lisbon, Portugal; ^3^Instituto de Saúde Ambiental (ISAMB), Faculdade de Medicina, Universidade de Lisboa, Lisbon, Portugal; ^4^Nursing Research, Innovation and Development Centre of Lisbon (CIDNUR), Nursing School of Lisbon, Lisbon, Portugal; ^5^Laboratório Associado TERRA, Faculdade de Medicina, Universidade de Lisboa, Lisboa, Portugal

**Keywords:** public health data, political decisions, COVID-19, health policy, health planning

In December 2019, a cluster of pneumonia cases was reported in Wuhan, China. Eventually it was identified, and the genetic sequence was thereafter disseminated, confirming a novel coronavirus infecting humans. Within just a few weeks, its rapid spread took on pandemic proportions, affecting people's lives and daily routines. As of May 2022, the COVID-19 pandemic is still raging on, posing challenges worldwide.

From its beginning, this pandemic has brought unexpected changes to health care systems and new challenges for public health, health monitoring, and health surveillance, namely in terms of the necessary data for clinical decisions, resource management, and policymaking. Moreover, health care systems had to maintain their non-COVID-19 activity while simultaneously the unrelenting impact of this new disease.

The scientific world, too, was taken by a hurricane, and witnessed an impressive number of COVID-19-related publications in record time. As of the end of April 2022, PubMed, one of the most well-known databases containing biomedical scientific literature, retrieved more than 255,000 citations with “COVID-19” as the search term. Of those, 72,587 records also included a reference to “data,” revealing a large body of literature that likely involved the use of data to study COVID-19.

Projects like the Population Health Information Research Infrastructure (1) are, we believe, currently conducting literature reviews to better understand the uses, the pathways, and the needs of population health data in these pandemic times. It will take years, or even decades, to understand exactly what happened and what lessons we must assimilate to prepare for similar health crises and take with us into our new day-to-day.

The primary aim of our Research Topic *Public Health Data Challenges of the COVID-19 Pandemic* was to focus on public health data in the contexts of worrying data deficits, emergent problems, and innovative solutions originating from the COVID-19 pandemic crisis. Nevertheless, it was the secondary aim—a focus on the solutions and knowledge brought by the pandemic—that received the most contributions. Curiously, most articles presented creative ways of acquiring data, thus failing to let us know more about the issues we were addressing.

The COVID-19 pandemic has exposed gaps that need to be addressed for society to return to a new normal that is better than before. Therefore, coordinated efforts among health care providers and public health officials at different levels are necessary—for example, to catch up on vaccination in the United States (2). The rapid development of COVID-19 vaccines has demonstrated that, with extensive data sharing, it is possible for researchers who have the necessary resources and novel technologies to conduct and apply their research throughout the response to the COVID-19 pandemic (3). This worldwide response has shown the importance of vigilance and preparedness for any infectious disease.

Policy approaches can be a highly effective tool for preventing exposure to other communicable disease outbreaks and protecting the health of the public. Researchers and public health leaders can examine the bidirectional effects of laws and policies, which are important levers to improve health and wellbeing. It is also crucial to measure and monitor the effect of laws and policies on the health status of populations. In this regard, policy interventions and assessments should examine the degree to which such interventions achieve equity, contribute to the effectiveness of health promotion programs, and shape the behaviors of various sectors that influence population health (2, 4).

We also ended up with interesting new perspectives on the effects of COVID-19 on hospital activity, particularly the need to balance usual care with addressing the needs of the pandemic. As an example, Kalanj et al. discuss the considerable reduction in hospital admission rates for non-COVID-19 health concerns, including services related to cardiovascular and malignant diseases.

Many case studies in this Research Topic focused on strategies employed by the scientific community for sharing their experiences and challenges in dealing with the ongoing pandemic situation, as well as strategies for providing policy recommendations and advice to governments on the impact of the disease. Additionally, once the COVID-19 vaccine became available, we received literature describing the challenges of vaccination management and inequitable distribution in many different countries. The articles were related to policies and strategies concerning public health, health policy, and health planning, with a focus on local, regional, and national approaches. The articles also applied a range of different empirical and conceptual approaches to the topics they covered. They explored topics such as health policy on supporting the first-dose vaccination campaigns that prioritized administration in the elderly (Pontrelli et al.) and even the effects of a delay in vaccine acquisition that slowed down a national vaccination program with the potential to accelerate the spread of the virus and lead to a new lockdown (Kamran and Ali).

Public health strategies such as containment and mitigation were emphasized in several articles. These articles also stressed the importance of evaluating publications and using meta-analyses for recommendations, even during a pandemic (Boudesseul et al.); modeled the effects of public health measures during the COVID-19 pandemic (Qiu et al.); discussed the effects of epidemic prevention and control (Chen et al.); and supported with data the effects of some specific measures such as school closures (Chao et al.).

Several review articles also focused on the importance of equity. For instance, Ruiz-Hornillos et al. examined the equity guarantees and transparency in decision-making processes, and Haring et al. explored the concept of trust in current and future equitable relationship-building between communities and public-private interests.

Although the pandemic was a new and challenging health problem, the increase in scientific publications during this period is likely related to the fact that more than a few journals fast-tracked COVID-19 research (5). Even so, COVID-19 brought to light the need for more robust public health data: one of the top three medical subjects in these publications was public health (6). Nonetheless, evidence is still lacking on the efficiency of healthcare systems regarding their efforts to address the indirect impacts of the pandemic and maintain their regular level of services, including their responsiveness to the demand for both critical medical services and non-elective procedures (Kalanj et al.).

More research is needed to establish public health policy data devoted to an emergency during a pandemic, and also to support alert systems that may contribute to increased preparedness for future pandemics.

The challenge remains to reinforce the need for more analysis and exploration of the situations where public health data is not available for informing political decisions; where information systems run into ethical barriers; and where new and innovative solutions had to be created to overcome the data needs imposed by the unexpected challenges of COVID-19 that occurred at local, regional, national, and international levels.

But, most importantly, we cannot keep perpetually reinventing the wheel in terms of the use of data for research, for population health, for public health, or for health monitoring and surveillance. We need to start talking openly about data. We must be able to build on each other's efforts, to share and improve data. We are not doomed to start anew for every single study or challenge—research is not a Sisyphean task!

## Author contributions

CF, PN, RF-S, and AC have worked on the conceptualization, methodology, and writing. All authors contributed to the article and approved the submitted version.

## Conflict of interest

The authors declare that the research was conducted in the absence of any commercial or financial relationships that could be construed as a potential conflict of interest.

## Publisher's note

All claims expressed in this article are solely those of the authors and do not necessarily represent those of their affiliated organizations, or those of the publisher, the editors and the reviewers. Any product that may be evaluated in this article, or claim that may be made by its manufacturer, is not guaranteed or endorsed by the publisher.
